# Pulsed CO_2_ Laser-Fabricated Cascades of Double Resonance Long Period Gratings for Sensing Applications

**DOI:** 10.3390/mi16080959

**Published:** 2025-08-20

**Authors:** Tinko Eftimov, Sanaz Shoar Ghaffari, Georgi Dyankov, Veselin Vladev, Alla Arapova

**Affiliations:** 1 Photonics Research Center, Université du Québec en Outaouais, Rue 101 St-Jean Bosco, Gatineau, QC J8X 3G5, Canada; alla.arapova.ca@gmail.com; 2 Central Laboratory for Applied Physics, Bulgarian Academy of Sciences, 61 Sanct Peterburg Blvd, 4000 Plovdiv, Bulgaria; ge.dyankov@gmail.com; 3 Department of Electrical and Computer Engineering, University of Alberta, 116 St & 85 Ave, Edmonton, AB T6G 2R3, Canada; shoargha@ualberta.ca; 4 Institute of Optical Materials and Technologies “Acad. J. Malinowski” (IOMT), Bulgarian Academy of Sciences (BAS), 109 “Acad. G. Bonchev” Str., 1113 Sofia, Bulgaria; 5 Department of Mathematics, Physics and Information Technologies, Faculty of Economics, University of Food Technologies, 26 Maritsa Blvd., 4002 Plovdiv, Bulgaria; v_vladev@uft-plovdiv.bg

**Keywords:** cascaded LPGs, turn around point LPGs, double resonance LPGs, fiber sensors, Mach Zehnder interferometer, CO_2_ fabrication of LPGs, biosensing

## Abstract

We present a detailed theoretical and experimental study of cascaded double resonance long period gratings (C DR LPGs) for fabricated sensing applications. The matrix description of cascaded LPGs is presented, and several important particular cases are considered related to the regular and turn around point (TAP) gratings. A pulsed CO_2_ laser was used to fabricate ordinary and cascaded DR LPGs in a photosensitive optical fiber. The responses of the fabricated C DR LPGs to surrounding refractive index (SRI) temperature as well to longitudinal strain have been studied. A statistical comparison of the SRI sensitivities of ordinary and cascaded DR LPGs is presented to outline the capabilities and advantages of cascaded DR gratings. It was experimentally established that the temperature dependence of the wavelength split at the TAP follows a logarithmic dependence and the sensitivity to temperature is inversely proportional to the temperature itself. We evaluate the temperature stability needed for SRI-based sensing application and the importance of fine-tuning to the operational point slightly after the TAP to ensure maximum sensitivity of the sensor.

## 1. Introduction

Long period optical fiber gratings (LPG) have been known for almost three decades [1,2,3], with the most straightforward application being single- and multi-parameter sensing [4,5]. Phase-shifted and cascaded LPGs were soon developed and shown to open new opportunities for a variety of sensing applications [6,7,8,9].

The theoretical description of the principle of operation of the LPGs is encapsulated in the mode coupling equations [2,7,9] both for weakly and strongly coupled LPGs [10]. The coupled mode equations describe the power exchange between the core fundamental mode and a higher-order cladding mode. The difference between the effective refractive indices of the two modes is wavelength-dependent. When the wavelength dependence is monotonous and the dispersion does not change sign, the LPG exhibits a single resonance wavelength for a particular pair of coupling modes. A particular case of a cascade of non-identical LPGs whose dispersion is with a constant sign has been analyzed theoretically and experimentally in [9]. When the dispersion changes sign over a specific spectral range, the LPGs exhibits a double resonance [11,12,13,14]. Since the two minima at the resonance wavelengths shift in opposite directions, double resonance (DR) LPGs exhibit a higher sensitivity to SRI, temperature and strain, which is why they have aroused an increased interest for a variety of sensing applications [15,16,17,18,19,20,21,22,23,24]. In the case of biochemical sensors, the DR LPGs have to be functionalized to achieve the desired selectivity.

Thus far, single DR LPGs are basically used for a large variety of biosensing applications, as explored in [13,15,18,21,22,23,24]. The LPGs are operated close to the turn around point (TAP), at which the single minimum splits and the grating becomes double resonance featuring a maximum sensitivity to SRI. However, while the sensitivity can be as high as 3000 nm/r.i.u., the minima are relatively broad and the accuracy of determining the positions of the minima, and hence the splitting, is lower.

To overcome the above problem, the performance of a cascade of two coated DR LPGs has been simulated theoretically [25], and it has been shown that much sharper resonance minima and a sensitivity to SRI as high as 12,550 nm/RIU can be achieved around a water refractive index of 1.33–1.334. Experimentally, cascaded DR LPGs have been fabricated using a 248 nm UV excimer laser in a PS1250/1500 photosensitive fiber, and their sensitivities to SRI and temperature have been measured. While the experimentally obtained minima are sharper, the measured sensitivities were still comparable (≤3000 nm/r.i.u.) to those of single DR LPGs.

In this paper we present the general matrix description of cascaded LPGs and the case of effective refractive difference varying monotonically and non-monotonically with wavelength, causing a split of the resonance that leads to a cascade of DR LPGs. Our objectives are (i) to compare the performances of single DR LPGs and cascaded DR LPGs, (ii) to study the techniques to fine-tune the cascaded grating to the TAP at a desired operational temperature and SRI; (iii) to measure the sensitivity to SRI, temperature and strain for future biosensing applications.

## 2. Matrix Description of LPGs and Cascaded LPGs

### 2.1. Coupling Matrices

#### 2.1.1. A Single LPG

**Figure 1:**
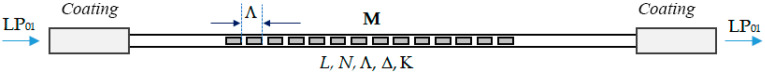
Schematic representation of a long period grating (LPG) of length *L*, containing *N* modulations of period Λ with normalized detuning Δ and coupling K coefficients.

A long period grating (LPG) is a series of periodic opto-geometrical modifications of a fiber, which cause the fundamental LP_01_ core mode to couple to a higher-order LP_0p_ cladding mode of the glass-surrounding medium waveguide. During fabrication the surrounding medium is air, and the effective waveguide is a glass–air waveguide. The fiber structural modifications can be introduced using different techniques such as refractive index modifications, geometrical deformations, mechanical stress and others. A schematic representation of an LPG is shown in Figure 1. The fundamental LP_01_ core mode with an amplitude $A_{c}^{o}$ is launched into the grating at the input.

As the mode propagates along the grating periodically, a fraction of its power is coupled to the higher-order *LP*_0p_ cladding mode. At the output of the grating, we have both modes with different amplitudes. The grating is described by a matrix **M,** which transforms the input vector $A^{o}$ of the mode electric fields into an output vector A as [2]:

(1) $$A(z)=M(z)\cdot A^{o},\;A^{o}=\left(\begin{array}{c} A_{c}^{o}\\ A_{cl}^{o} \end{array}\right),\;A=\left(\begin{array}{c} A_{c}\\ A_{cl} \end{array}\right)$$

where **A**_o_ and **A** are the input and output electric vectors containing electric field amplitudes $A_{c}^{o}$ and $A_{c}$ of the core LP_01_ and $A_{cl}^{o}$ and $A_{cl}$ of the cladding LP_0p_ mode. The matrix describing the coupling between the core and the cladding mode is [2]:

(2) $$M=\left[\begin{array}{cc} C+j\Delta S & jΚS\\ jΚS & C-j\Delta S \end{array}\right]\;\mathrm{where}\;\left[\begin{array}{c} C=\cos(\delta \beta z)\\ S=\sin(\delta \beta z) \end{array}\right]$$

In (2) *δβ* is the difference between propagation constant *β*_0_ the fundamental core mode (LP_01_) and *β*_0p_ of the higher-order cladding mode (LP_0p_), which is

(3a) $$\delta \beta =2\sqrt{\kappa^{2}+\delta^{2}}$$

where *κ* is the coupling and *δ* is the detuning coefficient between the core and cladding modes, as obtained by [2]:

(3b) $$\kappa =\frac{\pi }{2}\frac{\Delta \lambda_{0_{c}}}{\lambda^{2}}\Delta n_{eff}\;\mathrm{and}\;\delta =\pi \left[\frac{\Delta n_{eff}}{\lambda }-\frac{1}{\Lambda }\right]$$

The coupling (K) and detuning (Δ) coefficients from (2) are normalized to *δβ* as [2]:

(4) $$Κ=\frac{2\kappa }{\delta \beta },\;\Delta =\frac{2\delta }{\delta \beta },\;{Κ}^{2}+\Delta^{2}=1$$

The effective refractive index difference Δ*n*_eff_ depends on the wavelength (*λ*), temperature (*T*), the refractive index (*n*) of the surrounding medium and the fiber deformation (*ε*), and under fixed external parameters, the detuning parameter becomes zero at resonance wavelength, which is obtained from (3b) as:

(5a) $$\lambda_{c}=\Delta n_{eff}\Lambda$$

Thus, the resonance wavelength *λ*_c_ is dependent on *n*, *T* and *ε*, so

(5b) $$\lambda_{c}(n,T,\varepsilon )=\Delta n_{eff}(n,T,\varepsilon )\Lambda (T,\varepsilon )$$

which is the basis for a variety of sensing applications. With an LP_01_ mode at the input $A^{o}=(A_{c}^{o},0)$ and the LP_0p_ mode attenuated in the coated section of the fiber, only the fundamental LP_01_ mode is observed at the fiber output, with an amplitude *A*_c_(*z* = *L* = *NΛ*)

(6) $$A_{c}(L)=\left[\cos(\delta \beta L)+j\Delta \sin(\delta \beta L)\right]A_{c}^{o}$$

By letting *C* = cos(*δβL*) and *S* = sin(*δβL*), the measured intensity *I* is written as:

(7) $$I={\left|A_{c}(L)\right|}^{2}={\left|C+j\Delta S\right|}^{2}{\left|A_{c}^{o}\right|}^{2}=\left(C^{2}+\Delta^{2}S^{2}\right)I_{0}=\left(1-{Κ}^{2}S^{2}\right)I_{0}$$

The above-presented matrix description is valid in the case of uniform fiber, identical higher-order modes, identical periods and position independent coupling and detuning. Also, the grating length is *L* = *N*Λ. If these conditions are not met, the grating is non-uniform, and then it must be subdivided into *k* elementary sections, each one containing *N*_i_ periods and of length *L*_i_ = *N*_i_Λ. The coupling K_i_, the detuning Δ_i_ and the propagation constant difference in each of the subsections are constant but individual. In this case, the resultant matrix of the grating is the product of the matrices of each subsection **T** = **T**_k_·**T**_k−1_···**T**_2_·**T**_1_.

#### 2.1.2. A Cascaded LPG (C LPG)

**Figure 2:**
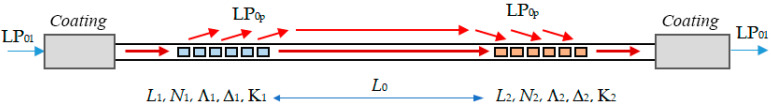
A cascaded LPG containing two different subgratings with parameters *L_i_*, *N_i_*, Λ*_i_*, Δ*_i_*, *K_I_*. *i* = 1, 2) separated by a fiber section of length *L*_0_ as an equivalent Mach–Zehnder modal interferometer between the fundamental LP_01_ core mode and the higher-order mode cladding LP_0p_.

A cascaded LPG (C LPG) consists of (at least) two gratings, LPG_1_ and LPG_2_, separated by a fiber section of length *L*_0_, whose transmission matrix is **T**_0_ (Figure 2) and describes the parallel independent propagation of the LP_01_ and LP_0p_ modes [7,9]:

(8a) $$A(z)=M_{2}(L_{2})\cdot T_{0}(L_{0})\cdot M_{1}(L_{1})\cdot A^{o}$$

(8b) $$M_{i}=\left[\begin{array}{cc} C_{i}+j\Delta_{i}S_{i} & j{Κ}_{i}S_{i}\\ j{Κ}_{i}S_{i} & C_{i}-j\Delta_{i}S_{i} \end{array}\right]\;(i=1,2),\;T_{0}=\left[\begin{array}{cc} a_{0}e^{-j\Delta \varphi } & 0\\ 0 & ae^{j\Delta \varphi } \end{array}\right]$$

In (8) it is assumed that each of the gratings is uniform. Physically, the CLPG (Figure 2) is a Mach–Zehnder interferometer, in which the LP_01_ mode which is well confined in the fiber core stands for the reference arm, while the higher-order *LP*_0p_ cladding mode which is more sensitive to external perturbations is the sensing arm. In a classical interferometer, the change in phase difference between the two arms causes the rise and displacement of new interference fringes. For a cascaded DR LPG (C DR LPG) the fringes will be manifested as an additional modulation in the spectral domain. Since in the general case the gratings are considered different, each one is characterized by its parameters *L_i_*, *N_i_*, Λ_i_, Δ_i_, K_i_ (*i* = 1, 2) [9]. In the intermediate section with length *L*_0_, the coupling coefficient is *κ*_0_ = 0 while Λ →∞ so *δ* = πΔ*n*_eff_/λ, so

(9) $$\delta \beta_{0}=\Delta n_{eff,0}\frac{2\pi }{\lambda }$$

and the accumulated phase difference between the modes (in (8b)) is

(10) $$\Delta \varphi =\delta \beta_{0}L_{0}/2=\Delta n_{eff,0}\frac{\pi }{\lambda }L_{0}$$

Also, in (8b), *a*_0_ and *a* are amplitudes of the core LP_01_ and the cladding LP_0p_ mode, the accumulated phase difference between which is

Assuming only core mode at the input and at the output, i.e., $A^{o}=(A_{c}^{o},0)$ and $A=(A_{c},0)$, after some manipulations the resultant intensity is obtained from (8) as [9]:

(11a) $$I=I_{0}a_{0}^{2}\left\{{\left[(R_{-}-\eta R)C_{\Delta \phi }+\eta R_{+}S_{\Delta \phi }\right]}^{2}+{\left[R_{+}C_{\Delta \phi }-(R_{-}+\eta R)S_{\Delta \phi }\right]}^{2}\right\}$$

where

(11b) $$R=K_{1}K_{2}S_{1}S_{2},\;R_{-}=C_{1}C_{2}-\Delta_{1}\Delta_{2}.S_{1}S_{2},\;R_{+}=\Delta_{1}S_{1}C_{2}+\Delta_{2}C_{1}S_{2}$$

and *η* is the ratio of the cladding-to-core mode ratio *η* = *a*/*a*_0_.

In the case of no intermediate section, *a*_0_ = *a* = 1 and Δ*ϕ* = 0.

(12) $$I(\lambda )=I_{0}\left\{{\left[C_{1}C_{2}-(\Delta_{1}\Delta_{2}+K_{1}K_{2})S_{1}S_{2}\right]}^{2}+{\left(\Delta_{1}S_{1}C_{2}+\Delta_{2}C_{1}S_{2}\right)}^{2}\right\}$$

which is the equation for an LPG of two non-identical parts.

In case the two parts are identical, *L*_1_ = *L*_2_ = *L*_0_ = *L*/2, *C*_1_ = *C*_2_ = *C*_0_ = cos(*δβL*_0_), *S*_1_ = *S*_2_ = S_0_ = sin(*δβL*_0_), Δ_1_ = Δ_2_ = Δ, K_1_ = K_2_ = K and, taking into account the normalization (4) as well as that, we obtain

(13a) $$I(\lambda )=\left\{{\left(C_{0}^{2}-S_{0}^{2}\right)}^{2}+\Delta^{2}{\left(2S_{0}C_{0}\right)}^{2}\right\}I_{0}=\left[\cos^{2}(2\delta \beta L_{0})+\Delta^{2}\sin^{2}(2\delta \beta L_{0})\right]I_{0}$$

(13b) $$I(\lambda )=\left(C^{2}+\Delta^{2}S^{2}\right)I_{0}=\left(1-K^{2}S^{2}\right)I_{0}$$

which is the result for an LPG with *L* = 2*L*_0_ from (7).

Using the general expression (11), different particular cases can be studied and simulated. Intentionally [5] or not, during fabrication, the LPGs may be non-uniform, and the simplest approach to account for existing non-uniformities is to assume that the two LPGs are different, though slightly.

### 2.2. Particular Cases

#### 2.2.1. Non-Uniform and Uniform Structures

In the case when the two LPGs are identical, Δn_eff1_ = Δn_eff2_ = Δn_eff_, *N*_1_ = *N*_2_ = *N* and Λ_1_ = Λ_2_ = Λ, and the same holds for the coupling and detuning coefficients. The structure is then uniform.

For specific applications during the simultaneous measurement of average temperature and thermal gradient [5,9], the cascade or the grating can be intentionally made non-uniform by either writing Λ_1_ ≠ Λ_2_ and Δn_eff1_ = Δn_eff2_ = Δn_eff_ or Λ_1_ = Λ_2_ = Λ and Δn_eff1_ ≠ Δn_eff2_. Differences in the Δn_eff_ can be achieved either by partially etching one of the subgratings, or by changing the intensity of the laser writing the grating. Such differences, though not constant, can be obtained during fabrication due to laser instabilities, non-uniform additional coatings, etc.

#### 2.2.2. Effect of Dispersion

The effective refractive index difference Δn_eff_ is wavelength-dependent, and we can distinguish two important cases: linear dependence with a constant dispersion, and non-linear dependence with a change in the magnitude and the sign of the dispersion:

(A) Linear dependence

**Figure 3:**
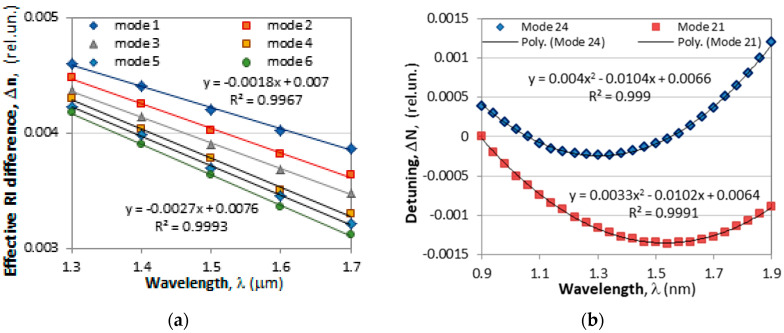
Effects of dispersion and the corresponding fits: (**a**) linear dependence of the effective refractive difference Δn_eff_ between the fundamental HE_11_ and six higher-order HE_1m_ modes on the wavelength [3]; (**b**) non-linear spectral dependence exhibiting a minimum of the detuning ΔN between the fundamental guided and two higher-order cladding modes [19].

In the linear case, we assume that over a certain spectral range, the effective refractive index Δn_eff_ is a linear function of wavelength in the form

(14) $$\Delta n_{eff}(\lambda )=A\lambda +B$$

where *A* and *B* are some constants depending on the particular mod, ***A*** being the dispersion illustrated by the examples in Figure 3 below as recovered from [3]. The detuning parameter (3b) can then be represented as:

(15a) $$\delta =\pi \left[\frac{\Delta n_{eff}}{\lambda }-\frac{1}{\Lambda }\right]=\pi \left[\frac{A\lambda +B}{\lambda }-\frac{1}{\Lambda }\right]=\pi \left[\frac{B}{\lambda }+A-\frac{1}{\Lambda }\right]$$

At resonance δ = 0 at a wavelength λ_c_, which is from (15a), it is related to the period as:

(15b) $$\Lambda =\frac{1}{A+\frac{B}{\lambda_{c}}}$$

As an example, for mode 1 from Figure 3a, *A* = −0.0018 μm^−1^ and *B* = 0.007. So, a resonance at λ_c_ = 1.5 μm would require a grating period of Λ = 348.4 μm.

The linear dependence of the type (14) has been taken into account for the analysis of the temperature behavior of cascaded LPGs made of non-identical LPGs [9].

(B) Non-linear dependence and double resonance LPGs

In this case we consider that for a specific higher-order cladding mode over a certain spectral range, Δ*n*_eff_ is non-linear and exhibits a minimum. As an example, we consider the detuning ΔN between the fundamental core and a higher-order cladding mode defined in [19] as:

(16) $$\Delta N=\Delta n_{eff}-\frac{\lambda }{\Lambda }$$

Figure 3b reproduces the wavelength dependences of Δ*N*(λ) as calculated in [19], which can be sufficiently well approximated (*R*^2^ = 0.999) by a quadratic function in the form

(17a) $$\Delta N(\lambda )=\Delta n_{eff}-\frac{\lambda }{\Lambda }=a\lambda^{2}+b\lambda +c$$

With reference to (16), the detuning coefficient δ from (3b) is found in this case to be wavelength-dependent, as:

(17b) $$\delta (\lambda )=\pi \left[\frac{\Delta n_{eff}(\lambda )}{\lambda }-\frac{1}{\Lambda }\right]=\frac{\pi }{\lambda }\left[\Delta n_{eff}-\frac{\lambda }{\Lambda }\right]=\frac{\pi }{\lambda }\Delta N(\lambda )$$

The resonance is then observed for wavelengths for which δ(λ) = 0, i.e., ΔN(**λ**) = 0, as follows from (17b). Taking the second order polynomial approximation (17a), we have:

(18a) $$\Delta N(\lambda )=\Delta n_{eff}-\frac{\lambda }{\Lambda }=a\lambda_{c}^{2}+b\lambda_{c}+c=0$$

or

(18b) $$\lambda_{c1,2}=\frac{-b\pm \sqrt{b^{2}-4ac}}{2a}$$

The spectral separation Δλ between the two resonance wavelengths λ_c2_ and λ_c1_ is

(18c) $$\Delta \lambda_{c}=\lambda_{c,1}-\lambda_{c,2}=\frac{\sqrt{b^{2}-4ac}}{a}$$

while the wavelength at which the detuning Δ*N*(*λ*) has a minimum is obtained by zeroing the first derivative of (18a) with respect to *λ*:

(18d) $$\lambda_{m}=-\frac{b}{2a}$$

The fitting coefficients *a*, *b* and *c* depend on the particular mode, the strength of the coupling, cladding diameter, the ambient temperature, the SRI, the imposed strain and other factors.

If we take the fitting coefficients for modes 24 and 21 from Figure 3b as examples, we can find using Equation (18) that

λ_c1_ = 1.5 μm, λ_c2_ = 1.1 μm, Δλ_c_ = 0.2 μm and λ_m_ = 1.3 μm   (mode 24)

λ_c1_ = 2.215 μm, λ_c2_ = 0.876 μm, Δλ_c_ = 1.34 μm and λ_m_ = 1.545 μm (mode 21)

As the coefficient *c* in (18a) shifts the parabolic function above or below the zero line for a certain value of *c*, the separation Δλ_c_ = 0 from (18c) is found to be fulfilled for

(19) $$c_{TAP}=\frac{b^{2}}{4a}$$

In this case, λ_c1_ = λ_c2_ = λ_m_ and is referred to as the turn around point (TAP). For *c* < *c*_TAP_ the LPGs exhibit two minima which diverge as *c* decreases, and the grating becomes double resonance grating (DR LPG).

## 3. Experimental Section

### 3.1. Experimental Setup

**Figure 4:**
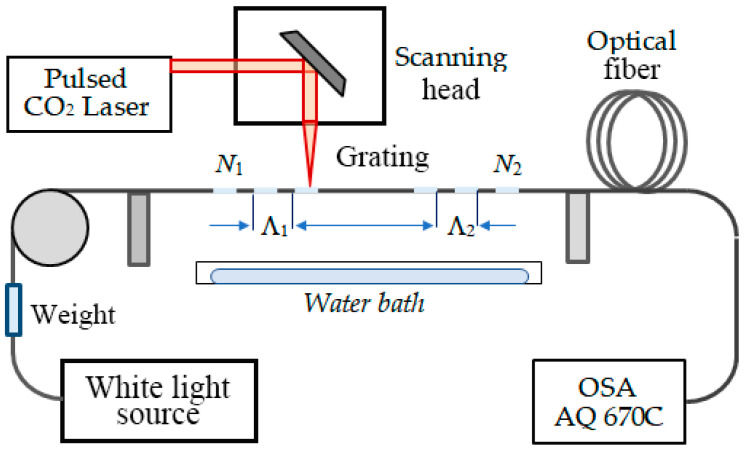
Experimental setup to fabricate LPGs.

The DR LPGs and the cascaded DR LPGs were fabricated using a pulsed CO_2_ laser (SYNRAD FHFL30-U) using the experimental arrangement presented schematically in Figure 4. The optical fiber was a photosensitive fiber PS1250/1500 (Fibercore) with a 9.6 μm fundamental mode field diameter. The C DR LPGs consisted of two parts with the same number of periods *N*_1_ = *N*_2_, the same period Λ = 207.6 μm and effective refractive indices Δn_eff,i_ (*i* = 1, 2). The DR LPGs were labeled as P xxx, implying the fiber is photosensitive, while the C DR LPGs as CP xxx, implying that the DR LPG is cascaded and written in a photosensitive fiber. The laser power expressed as a percentage of the maximum power sufficient to write the gratings, P_max_ = 30 W, was in the range between 13.1% and 14.4%. The scanning speed was 24 cm/s. Theoretically, the uncertainty in the total length is about 10 μm, which is determined by the laser marking software. This ensures a precision better than 0.1 μm in the period of the gratings. A K- broadband light source was used to observe the spectrum in the 400 nm to 1700 nm range of the OSA (AQ 6370C).

### 3.2. CO_2_ Laser Writing Process

Two possibilities exist for pulsed CO_2_ laser writing procedures. The first is to move the grating with a precision motorized translation stage while the laser beam remains fixed. In this case, the laser beam waist is stable, and the stability of the grating period depends on the translation stage. The second is to scan the laser beam along a fixed fiber section in which case the laser beam waist at the center of the grating will be different from that at the extremities. In both cases the grating is written in the air, but the final result is observed after the grating is placed in the immersion liquid, i.e., in water. This creates certain uncertainties, because the temperature of the water matters. Also, it is desirable to stop the writing process after the total number *N* of periods is completed.

We have used the second approach in our experiments. As the final spectrum of the grating in water varies due to random factors, it is necessary to fine-tune it to the initial position of being slightly split.

#### 3.2.1. Fabrication Procedure

The fabrication procedure was as follows:

(i) The particular grating pattern was drawn using the built-in software.
(ii) A section of about 10 cm of photosensitive PS1250/1500 fiber was cleaved, spliced in between SMF-28 lead-in/lead-out fibers and stripped bare over the whole length.
(iii) The fiber was placed and fixed to one of the holders and kept straight with a small weight of ≈3 g over a pulley at the other end.
(iv) The writing process was performed in air until the preprogrammed number of periods *N* was completed, which is considered as a writing scan.
(v) The changes in the spectrum were controlled during the writing process, which was carried out as consecutive scans if a single scan at a particular relative power was not sufficient to achieve the desired results. It should be noted that in air, the spectrum has a slight dip and is away from splitting.
(vi) After each scan, the LPG was immersed in the water bath to check if the LPG splits in water.
(vii) The process continues with the next scan until the LPG spectrum splits in water.

Depending on the laser power, the grating usually slightly splits in water after the first writing scan. After the writing process, the grating is placed in a measurement setup equipped with thermoelectric coolers, thermocouples to control the temperature. A U-shaped mini container is used for water or mixtures of water and glycerine having different refractive indices. The spectral response of the grating in water is measured.

In Table 1 in the Section Responses and Sensitivities to SRI below, which lists the SRI sensitivities of each grating, we have also provided data on the cascaded DR LPGs (*N* and *L*_0_) as well as the number of scans *n* times the power *p*, i.e., *n* × *p*, at which it was written. A scan usually lasts about a minute. Since the numbering of the gratings CP XXX follows the real sequence of fabrication, we notice that the power had to be increased in order to achieve a good result with one scan. From this observation we conclude that the laser power was not constant all the time, and this may be the cause for the statistical dispersion of the sensitivities.

#### 3.2.2. Fine-Tuning Procedure

As the scanning during the writing process is not interrupted, in water, the DR LPG may be split more than desired. To return it close to the TAP at a desired temperature (room temperature, for example) when immersed in a liquid with a desired refraction index (typically water), we used etching in a 10% solution of HF acid. A U-shaped semicircular container was placed upon a thermoelectric cooler (TEC) with attached thermocouples, which allowed us to maintain a constant temperature during the etching process and to measure the temperature sensitivity.

**Figure 5:**
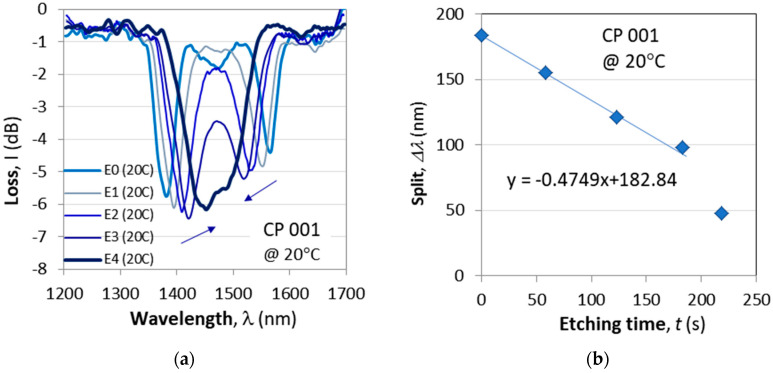

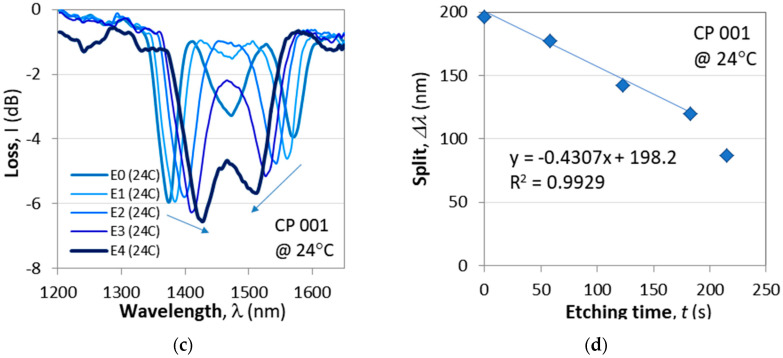
Fine-tuning of the C DR LPG CP001 to the TAP by means of etching in HF: (**a**) evolution of spectra at 20 °C; (**b**) reduction in spectral split vs. etching time at 20 °C; (**c**) same as (**a**) but at 24 °C; (**d**) same as (**b**) but at 24 °C.

The procedure is as follows:

(i) The spectrum of the DR LPG is measured in water.
(ii) The LPGs are then lifted from the U-shaped water container and are moved to a similar container filled with HF acid.
(iii) The grating is kept in the acid, and the spectrum is monitored continuously.
(iv) As the spectrum approaches the TAP, the grating is returned to the water bath upon the TECs.
(v) The temperature of the etched grating is varied to establish at what exact temperature the grating splits.
  The etching procedure typically yields a maximum fine-tuning rate of about a 0.5 nm/s decrease in the split, as is evident from Figure 5.

## 4. Results

### 4.1. Postfabrication Tuning

#### 4.1.1. Etching to TAP

As the process of fabrication is in air and the desired spectrum is observed in water, it is difficult to fine-tune the grating during the writing process. One of the reasons is that if the writing process is interrupted before completing the fixed number of periods, then the cascade will be of non-identical gratings. In the general case, if the desired initial spectrum is at turning point (TAP), then depending on the particular application, the operational ambient temperature, SRI and imposed longitudinal strain should also be known. We assume here that the desired initial operating conditions should be slightly split immediately after the TAP around room temperature (T = 22 °C) and in water (SRI ≈ 1.333). Figure 5a illustrates the transition from a widely split C DR LPG before etching (E 0) to the final spectral distribution of slightly split after the fourth etching (E 4), as measured at 20 °C as a function of the etching time. Figure 5c shows the spectra of the same grating at 24 °C. To the right in Figure 5b,d are the corresponding plots of the spectral split Δλ vs. the etching time.

The gratings that needed etching were CP 001, CP 003 and CP 004. The linear fits for CP 001 (Figure 5) show that the tuning rates are in the range from −0.431 nm/s to −0.475 nm/s; for CP 003, we find a rate of −0.918 nm/s, while for CP 004, the rate is −1.89 nm/s. These rates are quite different and were measured at somewhat different temperatures around 20 °C. The reasons for these differences need additional studies and statistics.

#### 4.1.2. Fine-Tuning to TAP by Means of Longitudinal Strain

**Figure 6:**
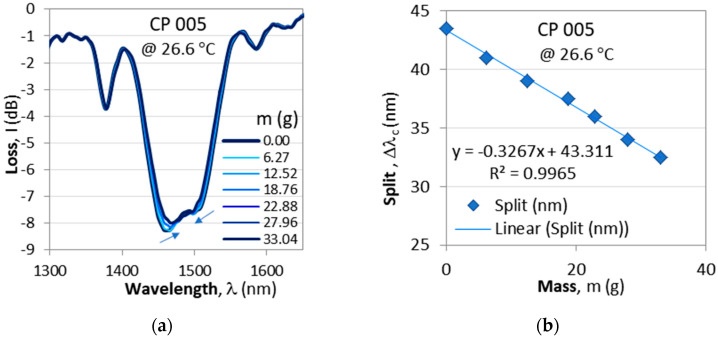
Fine-tuning to TAP by additional weights: (**a**) shrinking spectra; (**b**) wavelength split decreasing with the cumulative mass. Arrows indicate the direction of wavelength shifts.

For the purposes of sensing applications, the C DR LPG’s initial spectrum should be slightly split, because after the TAP the sensitivities are at a maximum. An additional fine-tuning to the point of discernible minima can be accomplished by means of a longitudinal strain which brings the minima closer to the TAP. Figure 6 presents the spectral changes in a non-etched cascaded DR LPG as it is subjected to an increasing longitudinal strain by weights with a mass increasing to about *m* = 33 g.

### 4.2. Responses and Sensitivities to the Measurands

In this section we study the responses of the DR LPGs and cascaded DR LPGs to SRI, temperature and strain.

#### Responses and Sensitivities to SRI

**Figure 7:**
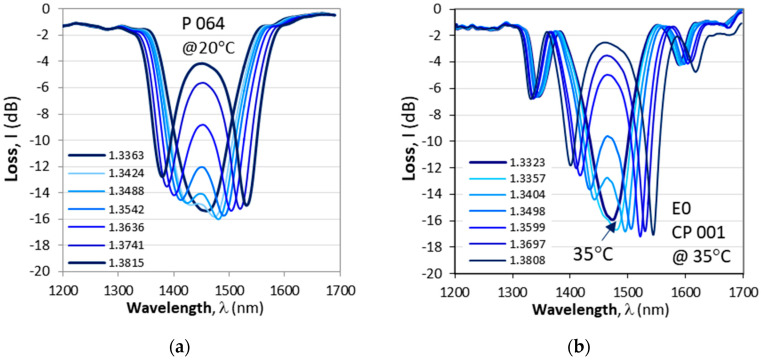
Evolution of spectra for a varying SRI of a single DR LPG and a cascaded DR LPG having the same period Λ = 207.65 μm: (**a**) DR LPG with *N* = 235 (*p* = 13.6%) splitting at 24.5 °C; (**b**) C DR LPG with *N*_1_ = *N*_2_ = 80 and *L*_0_ = 6 m. Arrows indicate the direction of wavelength shifts.

We start with the responses and the sensitivity to SRI changes, and in Figure 7 we show the responses to surrounding refractive index of a DR LPG (P 064) and a cascade of two DR LPGs (CP 001) at temperatures at which each one the gratings is just about to split at the TAP. Both gratings had the same period *Λ*. A brief comparison between the two responses yields the following observations:

(i) The cascaded DR LPG (CP 001) is narrower Δλ_0_ = 171 nm (E0) vs. ΔΛ = 260 nm for the DR LPG (P064). The spectral width Δλ_0_ above was measured prior to etching, but after etching it was 175 nm (E 1), 205nm (E 2 @ 9 °C), 175 nm (E 2 @ 48 °C), 181nm (E 3 @ 10.5 °C) and 177nm (E 3 @ 52.5 °C), and it essentially remains the same.
(ii) The minima of the cascaded DR LPG are narrower compared to those of the ordinary DR LPG.
(iii) The cascaded LPG exhibits additional minima because of the equivalent interferometer arrangements which shift at a lower sensitivity compared to the inner minima.

These observations mean that since the minima of the cascaded DR LPGs are sharper, this would increase the accuracy of the wavelength shifts. Also, the additional minima can be used for sensing purposes, though with a lower sensitivity.

The spectra of the gratings from Figure 7a,b were performed at different temperatures (20 °C for P 024 and 35 °C for CP 001) at which the observed splitting of the spectra occurs. This poses the question as to how the responses to SRI changes when etching is used to bring the TAP to a desired temperature.

**Figure 8:**
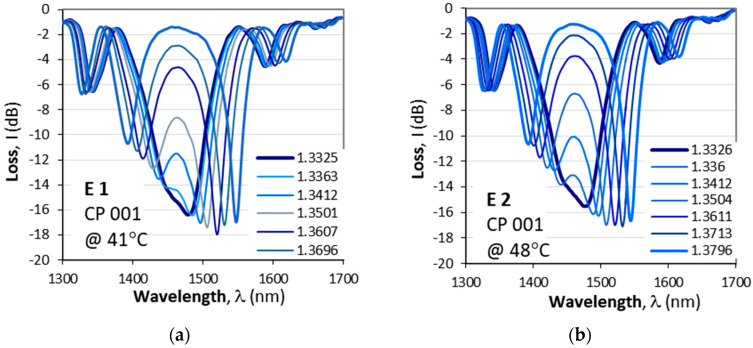

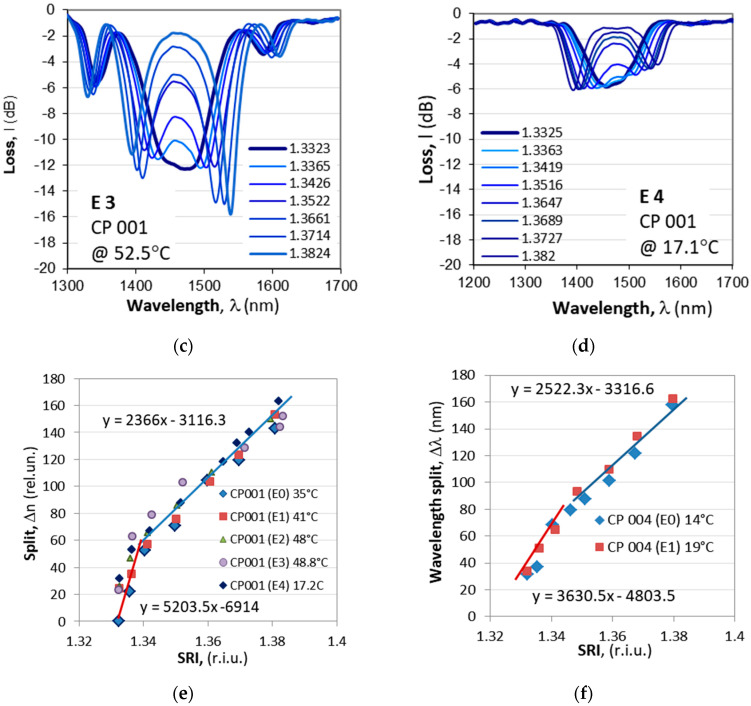
Evolution of spectra for a varying SRI of the cascaded DR LPG CP 001 (see Table 1): (**a**) after E 1 at 41 °C; (**b**) after E 2 at 48 °C; (**c**) after E 3 at 48.8 °C; (**d**) after E 4 at 17.2 °C; (**e**) all plots of Δn vs. SRI; (**f**) same as (**e**) but for CP 004.

Figure 8 illustrates the change in the spectrum and the measured wavelength splits vs. SRI after the corresponding etchings. Figure 8a–d summarize the spectral changes at the corresponding splitting temperature. After the fourth etching (E 4), the CP 001 cascade splits at two temperatures: 17.2 °C and 58 °C. Figure 8e summarizes the spectral splits Δλ vs. SRI for CP 001, while Figure 8f summarizes the splits for CP 004, which was fine-tuned after the first etching.

Table 1 below summarizes the measured sensitivities in the two distinct SRI ranges that are readily outlined in Figure 8e,f in the 1.33 to 1.35 interval and above 1.35.

As seen from Table 1, the sensitivities S(I) and S(II) of the two SRI intervals differ considerably, and a statistical variation is clearly observable. To evaluate the practical difference between a simple DR LPG and a cascade of DR LPGs, we performed some statistical analysis, and the sensitivities of the eleven cascaded DR LPGs are compared to the sensitivities of 25 DR LPGs whose spectra are similar to those from Figure 7a.

We have plotted the cumulative probability F* of the measured sensitivities *S*_n_ (n = 1 to N), with *N* = 12 for the C DR LPGs and *N* = 25 for the DR LPGs

(20) $$F*\left(S_{n}\right)=\frac{n-0.3}{N+0.4},$$

**Figure 9:**
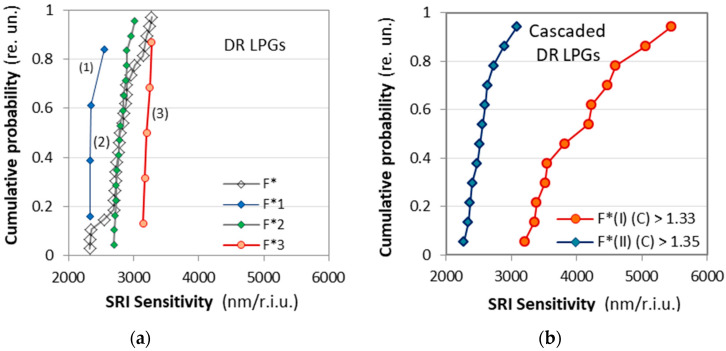
Cumulative probability distributions F* vs. SRI sensitivity *S*: (**a**) F* for 25 DR LPGs and their three subpopulations; (**b**) F* for the two subintervals of the 12 C DR LPGs.

The plots of the cumulative probability F* vs. the measured SRI sensitivity S for the single DR LPGs are shown in Figure 9a, while Figure 9b shows them for the C DR LPGs. The sensitivities for the DR LPGs were for the range ≥1.333, while those for the C DDR LPGs were for the two outlined in Figure 8d,e intervals. As is seen, the results for the DR LPGs (empty rhombs) in Figure 9a clearly indicate the existence of three subpopulations numbering 4, 16 and 5 samples centered correspondingly around different mean values, namely (1) around 2387.7 nm/r.i.u., (2) around 2809.8 nm/r.i.u. and (3) around 3208 nm/r.i.u. The cumulative probability distributions *F*_1_*, *F*_2_* and *F*_3_* for each of these subpopulations were calculated using (20) for *n* being 4, 16 and 5, and then plotted together for comparison. The average sensitivity over all subpopulations is 2821.9 nm/r.i.u. In comparison, the cascaded DR LPGs systematically exhibit a higher sensitivity S(I) over the initial SRI interval, followed by a lower sensitivity above 1.35—S(II), as listed for each grating in Table 1. Figure 9b presents the cumulative probability distributions F*(I) and F*(II) of these two sensitivities, plotted in the same scale as those of the simple DR LPGs (Figure 9a). Their averages are ≈2564 nm/r.i.u. for the lower sensitivity at higher SRI and ≈4000 nm/r.i.u. for the SRI around water.

The comparison of the statistical results as described by the cumulative probability distributions leads to the following observations:

(i) The average sensitivity of the simple DR LPGs is higher by about 9% than the average sensitivity of the cascaded DR LPGs for SRI > 1.35.
(ii) The sensitivity of the cascaded DR LPGs around water (SRI = 1.33 ÷ 1.35) is on average about 56% higher than the average sensitivity of the simple DR LPGs.
(iii) The variance of the sensitivities around lower SRI for the cascaded DR LPGs is quite large and can vary by up to 70%.

The above observations imply the following conclusions: First, cascaded DR LPGs are better suited for more sensitive measurements over a limited range of SRI changes around water. Second, individual calibrations must be performed for the grating used.

To understand the causes of the formation of subpopulations, several requirements are needed: fabricate gratings with the same parameters (*N*, *L*_0_ and the laser power *p*), which is easy; make sure the laser is stable during the writing procedure, which is more difficult; make sure that the laser beam-waist remains the same all along the grating length, which is also more difficult. As noted at the end of Section 3.2.1, the optical power and number of scans had to be increased for some gratings (see Table 1). We notice from Table 1 that the gratings written at a higher nominal (preset) power or with more scans have greater sensitivities. We take gratings from #1 to #5 from Table 1 whose power is ≤14.2% written with one scan (lower power group), and compare them with gratings #6 to #12 written at a power ≥ 14.2% and/or n ≥ 1 (higher power group). For SRI ≤ 1.35, we find the average sensitivities are ≈3735 nm/r.i.u. for the lower power group vs. ≈4310 nm/r.i.u. for the higher power group, while for SRI > 1.35 we have ≈2411 nm/r.i.u. vs. ≈2690 nm/r.i.u. Thus, the sensitivities obtained and the statistical dispersion correlate with the laser power level, laser stability and the number of scans.

### 4.3. Response and Sensitivity to Temperature

#### 4.3.1. Spectral Evolution

**Figure 10:**
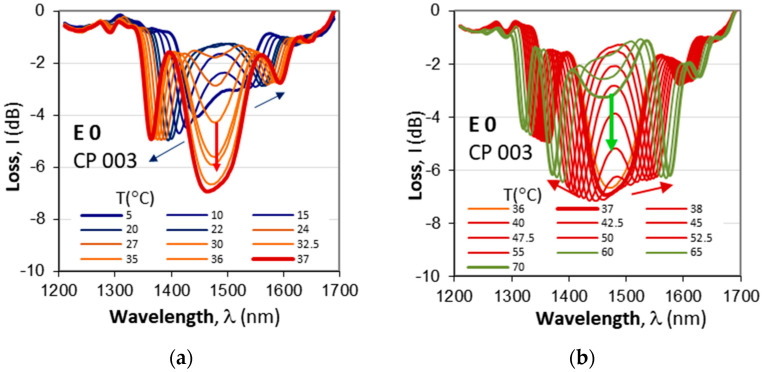
Evolution of spectra for a varying temperature in the 5 °C to 65 °C temperature range for cascaded DR LPG CP 003 with *N*_1_ = *N*_2_ = 115, *L*_0_ = 13 mm: (**a**) lower temperature range from 5 °C to 37 °C splitting at ≈36 °C; (**b**) higher temperature range from 36 °C to 65 °C and a new minimum appearing at 60 °C for a high-temperature split. The spectra suggest that a splitting occurs under 5 °C (around 0 °C), a second at 36 °C, and a third above 70 °C as summarized in Table 2.

We now consider the temperature responses of the cascaded LPGs and first note that in Figure 8, the responses to SRI changes at higher temperatures (Figure 8a–c) are deeper (around −17 dB) compared to the same responses at lower temperatures (17.1 °C). As the number of temperatures is about 20, we present the evolution in two stages: at lower and at higher temperatures. Figure 10a presents the results for CP 003 for temperatures from 5 °C, at which the spectra were already lightly split, until the first split at 37 °C. As is seen, under 30 °C, the depth of the cascaded DR LPG is between −3 dB to −5 dB, after which it drops to −7 dB as evidenced in Figure 10a,b.

**Figure 11:**
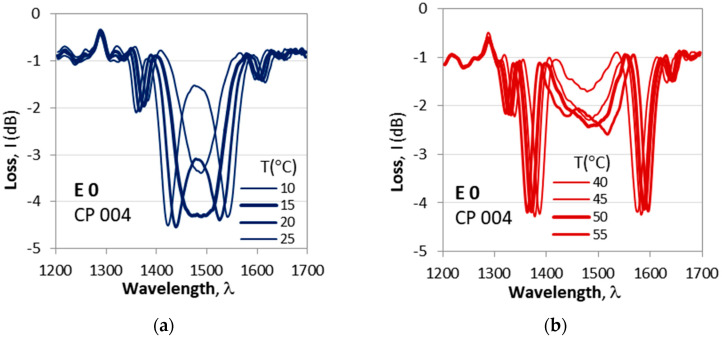
Evolution of spectra for a varying temperature in the 10 °C to 55 °C range for cascaded DR LPG CP 004 with *N*_1_ = *N*_2_ = 118, *L*_0_ = 13 mm: (**a**) lower temperature range from 10 °C to 25 °C splitting at ≈15 °C; (**b**) higher temperature range from 40 °C to 55 °C and a new minimum appearing at 50 °C for a high-temperature split.

Cascaded DR LPG CP 004 exhibits somewhat different behavior. The first observed split at 15 °C (Figure 11a) is deeper (−4.2 dB) compared to the next one at 50 °C (−2.3 dB) shown in Figure 11b, as summarized in Table 2.

**Figure 12:**
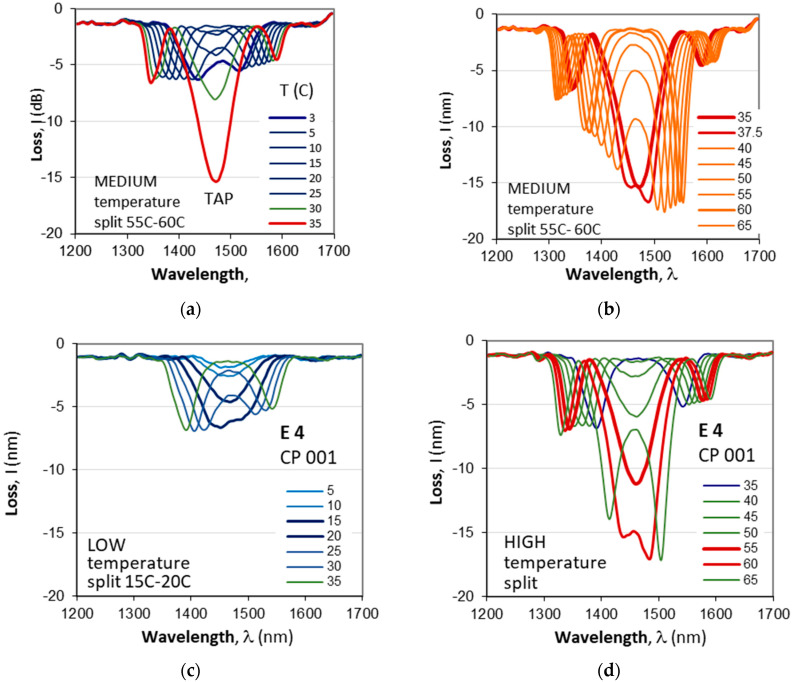
Temperature-induced evolution of the spectra of a C DR LPG CP 001 prior to etching (E0) and after 4th etching (E4): (**a**) a medium temperature split before etching at the extremity of the 5 °C–35 °C low-temperature range; (**b**) a spectral split before etching (E0) in the 35 °C–37.5 °C range within the 35 °C–65 °C interval; (**c**) low-temperature split after 4th etching (E4) around the middle of the 5 °C–35 °C range; (**d**) high-temperature split after 4th etching (E4) around the middle of the 40 °C–65 °C range.

Gratings CP 003 (Table 2) and CP 004 (Table 3) are weak and shallow compared to CP 001, which is deeper. To study these observations, we measured the evolution of the spectra of CP 001 vs. temperature changes before (E0) and after etching (E1, …, E4). Figure 12a–d show the temperature dependence before etching (E0) and after the last fourth etching (E4). We clearly see the effect of the abrupt increase in the grating depth at the TAP. Before etching, the TAP is at 35 °C, which is in the middle of the temperature range. Table 4 summarizes the parameters of the splits at lower and higher temperatures of CP 001, which confirms the observation. It implies that the detuning at higher temperatures is lower than that at lower temperatures over the range of interest.

#### 4.3.2. Sensitivity Curves

**Figure 13:**
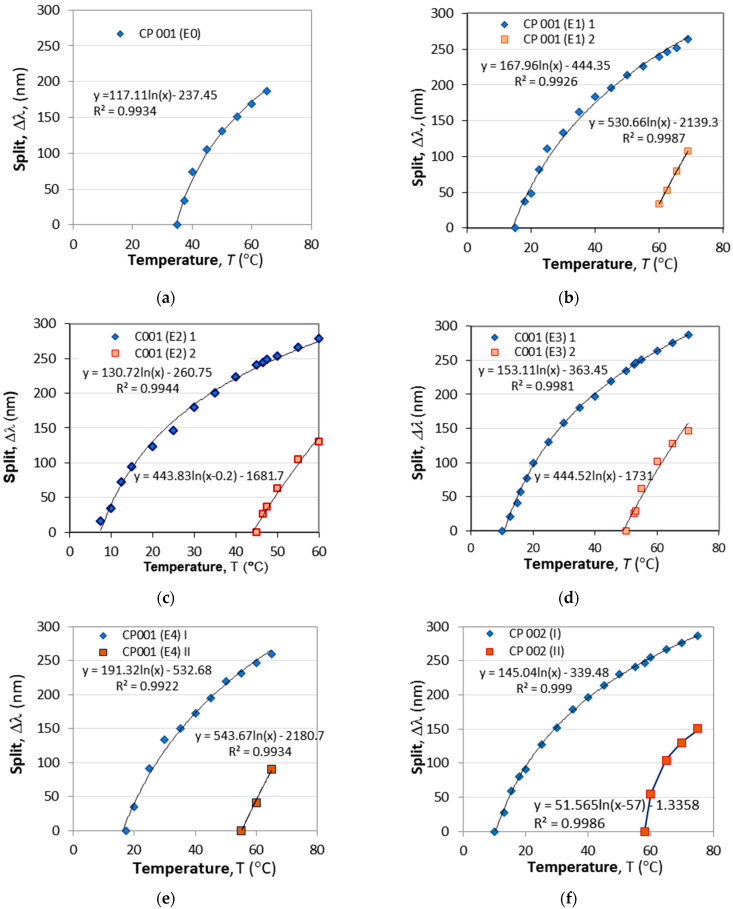
The effect of HF acid etching on temperature dependence of the wavelength splits: (**a**) non-etched grating (E0); (**b**) after E1; (**c**) after E2; (**d**) after E3; (**e**) after E4 and (**f**) non-etched CP 002.

We now study the temperature dependence of the wavelength split Δλ(*T*). Figure 13a–e present the temperature dependences of the split of CP 001 before etching (E0) and after each consecutive etching (E1, …, E4) as well of grating CP 002 (Figure 13f). The Δλ(*T*) at lower (blue rhombs) and at higher (red squares) temperatures is shown. As we see from the figures, the Δλ(*T*) curves are very well fit by a logarithmic dependence in the form:

(21) $$\Delta \lambda (T)=C\ln(T-T_{b})+C_{0},\;T\geq T_{\mathrm{TAP}}$$

In (21), *C* and *C*_0_ are coefficients, while *T*_b_ is a biasing temperature. Equation (21) is valid for temperatures above that of the turn a around point (TAP), i.e., T ≥ *T*_TAP_. The sensitivity to temperature S_T_(*T*) thus would be

(22) $$S_{T}(T)=\frac{d\left[\Delta \lambda (T)\right]}{dT}=\frac{C}{T-T_{b}}$$

and it is inversely proportional to temperature.

**Figure 14:**
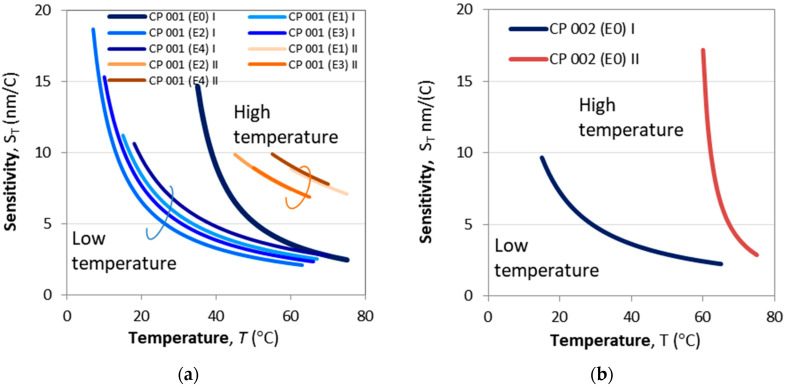
Temperature dependence of the sensitivity to temperature *S*_T_ calculated from (22): (**a**) sensitivities to temperature at lower and higher temperature splits after four consecutive etchings for CP 001; (**b**) sensitivity to temperature at lower and higher temperature splits for a non-etched CP 002.

Figure 14a,b show the sensitivities at lower and higher temperature splits for CP 001 and CP 002 calculated using (22) and the fitting parameters from Figure 13a–f. The plots in Figure 14a show that the etching process basically does not change the dependence, but simply shifts along the temperature scale.

The logarithmic fits were found to be well suited for all of the cascaded DR LPGs, and the results for the fitting parameters *T*_b_, *C*, *C*_0_, *T*_TAP_ and the sensitivity at *S*_TAP_ are listed in Table 5.

**Figure 15:**
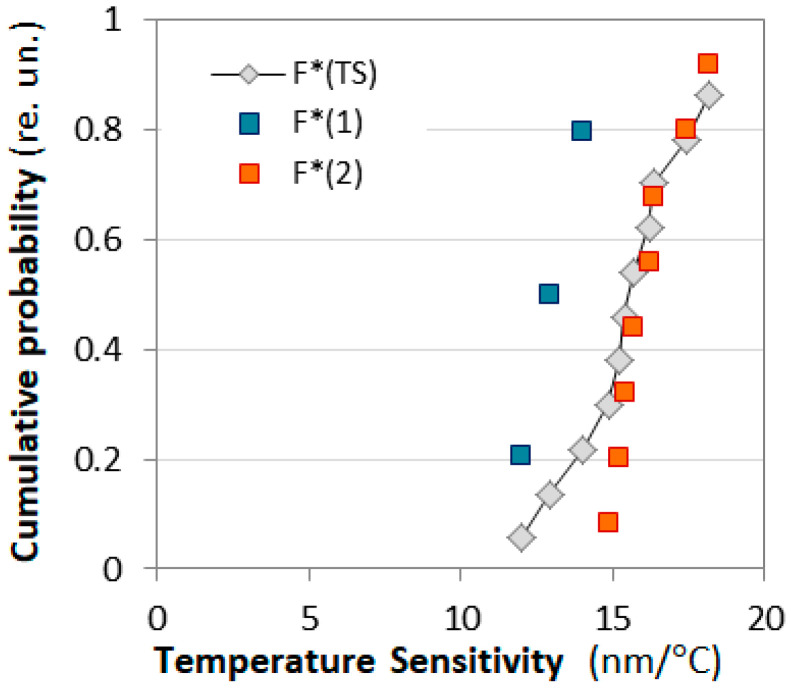
Cumulative probability distributions F* vs. temperature sensitivity T*S*.

With reference to Table 5, and Figure 15 we present a plot of the cumulative probability F*(TS) of the sensitivity to temperature around TAP temperature. The average sensitivity is S_T,avg_ ≈ 15.28 nm/°C, but we clearly see two subpopulations with cumulative functions F*(1) and F*(2) whose mean values are 12.97 nm/°C and 16.15 nm/°C. Thus, we see that most of the C DR LPGs (73%) are well grouped around 16.15 nm/°C. The gratings from the second subpopulation centered around 12.07 nm/°C are among the subpopulation of higher sensitivity to SRI listed in Table 1 and Figure 9b.

These results indicate that the highest sensitivity to temperature is observed at the TAP, which is important in the case of SRI sensing applications in which temperature stability must be maintained. As seen from Table 5 the temperature sensitivity varies between ≈12 nm/°C and ≈18 nm/°C theoretically at the TAP. As the gratings are used slightly after split, we assume that a meaningful value of 10 nm/°C and an instability of 0.1 °C would lead to a 1 nm fluctuation of the reading of the wavelength split, which will lead to an uncertainty of the SRI determination of 1/S_n_. For an average value S_n_ ≈ 4135 nm/r.i.u. (see Figure 9b) the SRI uncertainty would be ≈2.4 × 10^−4^. If the accuracy of wavelength shift determination is 0.1 nm, then the SRI uncertainty would be ≈2.4 × 10^−5^, which would require a temperature stability of 0.01 °C. Based on our experience in biosensing with DR LPGs [23,24], to ensure such a stability in SRI-based sensing applications, the following measures are recommended: perform the measurements in premises with temperature control and avoiding air flows; place the sensing grating in a massive and thermally isolated housing, ensuring a cover for the sensing area with a small volume around the grating in the liquid container; after changing the concentration of the liquid, wait for several minutes for the temperature to stabilize and vapors to saturate.

### 4.4. Sensitivity to Strain

**Figure 16:**
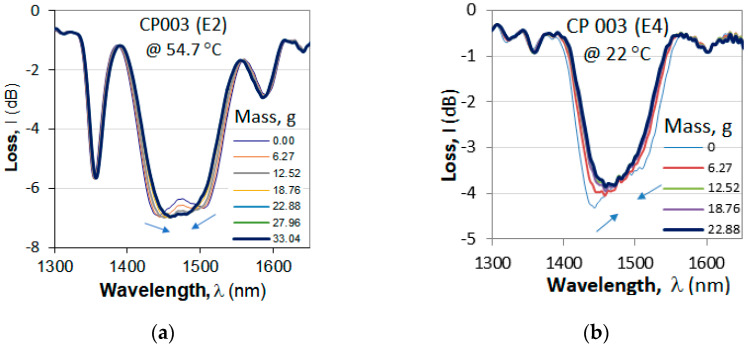

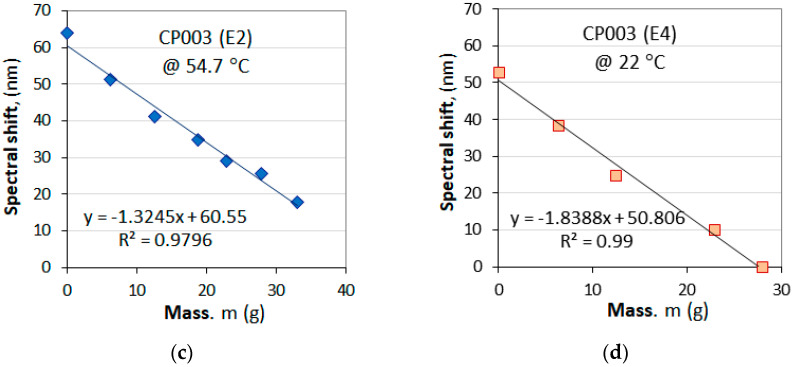
Fine-tuning to TAP of cascaded grating CP 003 caused by additional weights: (**a**) shrinking spectra after E2 at 54.7 °C; (**b**) shrinking spectra after E4 at 22 °C; (**c**) spectral shift vs. additional mass after E2; (**d**) spectral shift vs. additional mass after E4. Arrows indicate the direction of the shifts.

In Section 4.1.2, we showed that accurate fine-tuning to the TAP can be achieved by using additional weights to increase the strain along the grating. The sensitivity to strain, however, depends on the etching stage and the TAP temperature. Figure 16 presents the shrinking spectra of grating CP 003 after second (E2) and fourth (E4) etching. After E2, the split occurs around 55 °C, while after E4, the cascaded grating splits at around 22 °C. As is seen from Figure 16c,d, the sensitivities are different, and after E4, it is higher *S*_m_ = −1.8388 nm/g.

These results show that the sensitivity to strain is quite individual and should be measured for each grating fabricated for sensing applications.

## 5. Discussion

The cascaded DR LPGs fabricated and experimentally studied in this paper demonstrated advantages over plain DR LPGs. Their SRI sensitivity is higher at the TAP, which usually is at room temperature in the case of the medium being water (n ≈ 1.333), which is the case for most of the biosensing applications. However, for other sensing applications in which the medium is not water but blood with a refractive index of 1.3475 at 1.55 μm [26], the cascaded grating will have to be tuned to the TAP around a different value using the two methods described and used in this work.

The importance of fine-tuning becomes equally important in biosensing applications because in most cases, the outer surface of the gratings will have to be functionalized to become selective. Functionalization in most of the cases means the deposition of nanolayers of specific agents [18,21,22,23,24,25], which will increase the split and may change the sensitivity. The return to the TAP then can be best performed using only additional strain, which is easier to control than temperature.

As the sensitivity to temperature around the TAP is at a maximum, ambient temperature needs to be strictly controlled, and air currents suppressed.

The above statement means that fine-tuning using etching should be performed for a particular set of operational temperatures and SRIs.

The wavelength split is temperature-dependent and follows a logarithmic dependence; hence, the sensitivity to temperature is inversely proportional to the temperature itself.

Several issues remain for future studies.

First, a more systematic experimental investigation of the effect of integrating spacing *L*_0_ is needed.

Second, more statistical data is needed on the strain sensitivities.

Third, as the TAP is for a given set of SRIs, temperature and strain operational conditions, cross-sensitivity studies need to be performed. More precisely, as the cascaded DR LPGs are needed for SRI-based sensors, it would be of interest to measure the sensitivity to SRI at different levels of strain at room temperatures. Such measurements should reveal if strain-induced fine-tuning at a given temperature will affect the sensitivity to SRI and thus the sensitivity of the biosensor.

Lastly, it is worth comparing the pulsed CO_2_ laser writing technique with the other widely used techniques: UV excimer laser and femtosecond (fs) laser.

The UV excimer laser (KrF at 248 nm) usually makes use of phase masks which must be specially made for a particular period of the grating. This technique offers very repeatable gratings. There are, however, several shortcomings. The gratings are not thermally stable and have to be annealed, which will modify the TAP conditions. The laser is large, and the equipment is rather expensive. The phase masks are specific and custom made. As the UV introduces defects into the fiber to modify the refractive index, the mechanical strength of the grating is reduced, which would be a problem if it has to be under strain for a longer period of time.

The fs laser technique is a point-by-point technique, but as the focusing length is short, the whole fiber must be moved with a precision translation stage [27]. The technique offers a lot of flexibility and does not need annealing, as with the excimer lasers, and can be very repeatable once the proper parameters are established. The disadvantages are very expensive laser equipment, needing special maintenance and slower fabrication. For some reason, the SRI sensitivities fabricated by this method [27] are lower than those reported in this work.

Compared to the above methods, the pulsed CO_2_ laser technique is considerably lower cost, and the gratings are thermally stable and mechanically robust. The gratings will, however, have greater polarization-dependent losses, which would be an issue should polarization be important.

## 6. Conclusions

The presented results and analysis make it possible to formulate the following conclusions.

First, cascaded DR LPGs demonstrate a definite advantage over single DR LPGs in so much as a higher sensitivity to SRI at the TAP is concerned for applications to biosensing.

Second, the results of the increased SRI sensitivity as experimentally obtained in this work are significantly higher than those reported in the literature.

Third, around the TAP, the sensitivity to temperature is the highest (of the order of 10 nm/°C), which implies that to avoid thermally induced instabilities in SRI-based biosensors, the ambient temperature must be stabilized with variations less than 0.01, which is demanding.

Fourth, fine-tuning close to the TAP can easily be realized by manipulating the longitudinal strain. This is all the more needed in cases where the cascaded DR LPGs are functionalized with nanolayers for a desired sensing selectivity, which will additionally increase the split of the grating.

## Figures and Tables

**Table 1:** Comparison table of the sensitivities to SRI of the eleven cascaded DR LPGs.

#	Label	2 × N + L_0_	*n* × *p* (%) 30 W max	S(I) 1.33 to 1.35	S(II) > 1.35	@ T(°C)
1	CP 001	2 × 80 + 6 mm	1 × 13.3%	4182.7	2369.1	20
2	CP 004	2 × 118 + 13 mm	1 × 13.3%	3354.6	2273.3	22.5
3	CP 005	2 × 80 + 6 mm	1 × 13.3%	3518.4	2518	24.2
4	CP 013	2 × 118 + 13 mm	1 × 14.2%	4231.1	2342.3	25.5
5	CP 014	2 × 118 + 13 mm	1 × 14.2%	3385.4	2555.3	23
6	CP 015	2 × 118 + 13 mm	6 × 14%	4602.5	2399.1	22.5
7	CP 017	2 × 118 + 13 mm	1 × 14.2%	3549	2728.5	20.5
8	CP 018	2 × 118 + 13 mm	3 × 14%	5457.4	2634.1	25.5
9	CP 019	2 × 118 + 20 mm	1 × 14.3%	5063.2	2476.9	22.7
10	CP 020	2 × 118 + 20 mm	1 × 14.3%	3816.9	2599.3	20.5
11	CP 022	2 × 118 + 20 mm	1 × 14.4%	4475.2	2893.2	22
12	CP 023	2 × 118 + 20 mm	1 × 14.4%	3201.8	3090.2	25

**Table 2:** Evolution of the low- and high-temperature splits at a TAP wavelength of 1467 nm for a non-etched cascaded DR LPG CP 003 (*N*_1_ = *N*_2_ = 115, *L*_0_ = 13 mm).

Etching Stage	Low-Temperature Split	Mid-Temperature Split	High-Temperature Split
E 0 (prior to etching)	<5 °C (≈0 °C) @ −4 °C	36 °C @ −7 dB	>70 °C

**Table 3:** Evolution of the low- and high-temperature splits at a TAP wavelength of 1467 nm for a non-etched cascaded DR LPG CP 004 (*N*_1_ = *N*_2_ = 118, *L*_0_ = 13 mm).

Etching Stage	Low-Temperature Split	High-Temperature Split
E 0 (prior to etching)	15 °C @ −4.2 dB	50 °C @ −2.3 dB

**Table 4:** Evolution of the low- and high-temperature splits at a TAP wavelength of 1451.5 nm for cascaded DR LPG CP 001.

Etching Stage	Low-Temperature Split	High-Temperature Split
E 0 (prior to etching)	<0 °C	36 °C @ −14 dB
E 1 (after 1st etching)	5.2 °C @ −3 dB	41.1 °C @ −16 dB
E 2 (after 2nd etching)	10 °C @ −5.8 dB	46.5 °C @ −16 dB
E 3 (after 3rd etching)	12 °C @ −6.2 dB	52 °C @ −16 dB
E 4 (after 4th etching)	17.2 °C @ −6.5 dB	58 °C @ −16 dB

**Table 5:** Comparison table of the sensitivities to temperature of the eleven cascaded DR LPGs.

#	Label	T_b_ (°C)	C	C_0_	TAP Temperature (°C)	S_TAP_ (nm/°C)
1	CP 001	8.6	191.32	−296.53	20.8	15.68
2	CP 004	16.8	92.219	−124.2	22.5	16.18
3	CP 005	19.3	106.24	−174.79	25.8	16.34
4	CP 013	18.1	99.381	−167.9	24.8	14.83
5	CP 014	13.2	126.85	−275.26	23.8	11.97
6	CP 015	13.7	121.97	−244.41	22.4	14.02
7	CP 017	17.7	99.949	−154.49	23.2	18.17
8	CP 018	16.6	119.01	−237.53	25.8	12.94
9	CP 019	18	100.94	−154.28	23.8	17.40
10	CP 020	15	98.373	−154.45	21.4	15.37
11	CP 022	14	121.51	−247.33	22	15.19
12	CP 023	19.3	106.24	−174.79	25.8	16.34

## Data Availability

The data presented in this study are available on request from the corresponding author.
